# Supplementary material for: A male-specific doublesex isoform reveals an evolutionary pathway of sexual development via distinct alternative splicing mechanisms
Source: Commun Biol. 2022 Jul 22;5:728. doi: 10.1038/s42003-022-03664-7 (PMC9307624; doi:10.1038/s42003-022-03664-7)
Supplement: Supplementary file 2 — Supplementary Information [file 42003_2022_3664_MOESM2_ESM.pdf]

Supplementary Information

A male-specific *doublesex* isoform reveals an evolutionary pathway of sexual development via distinct alternative splicing mechanisms

Caihong Han [1]*, Qionglin Peng [1]*, Xiangbin Su [1], Limin Xing [1], Xiaoxiao Ji [1], Yufeng Pan [1,2,#]

[1]The Key Laboratory of Developmental Genes and Human Disease, School of Life Science and Technology, Southeast University, Nanjing, 210096, China.

[2]Co-innovation Center of Neuroregeneration, Nantong University, Nantong, 226019, China.

*These authors contributed equally to this work.

[#]Correspondence to: pany@seu.edu.cn (Y.P.)

This PDF file includes:

Supplementary Fig. 1-9 and legends

Supplementary Table 1

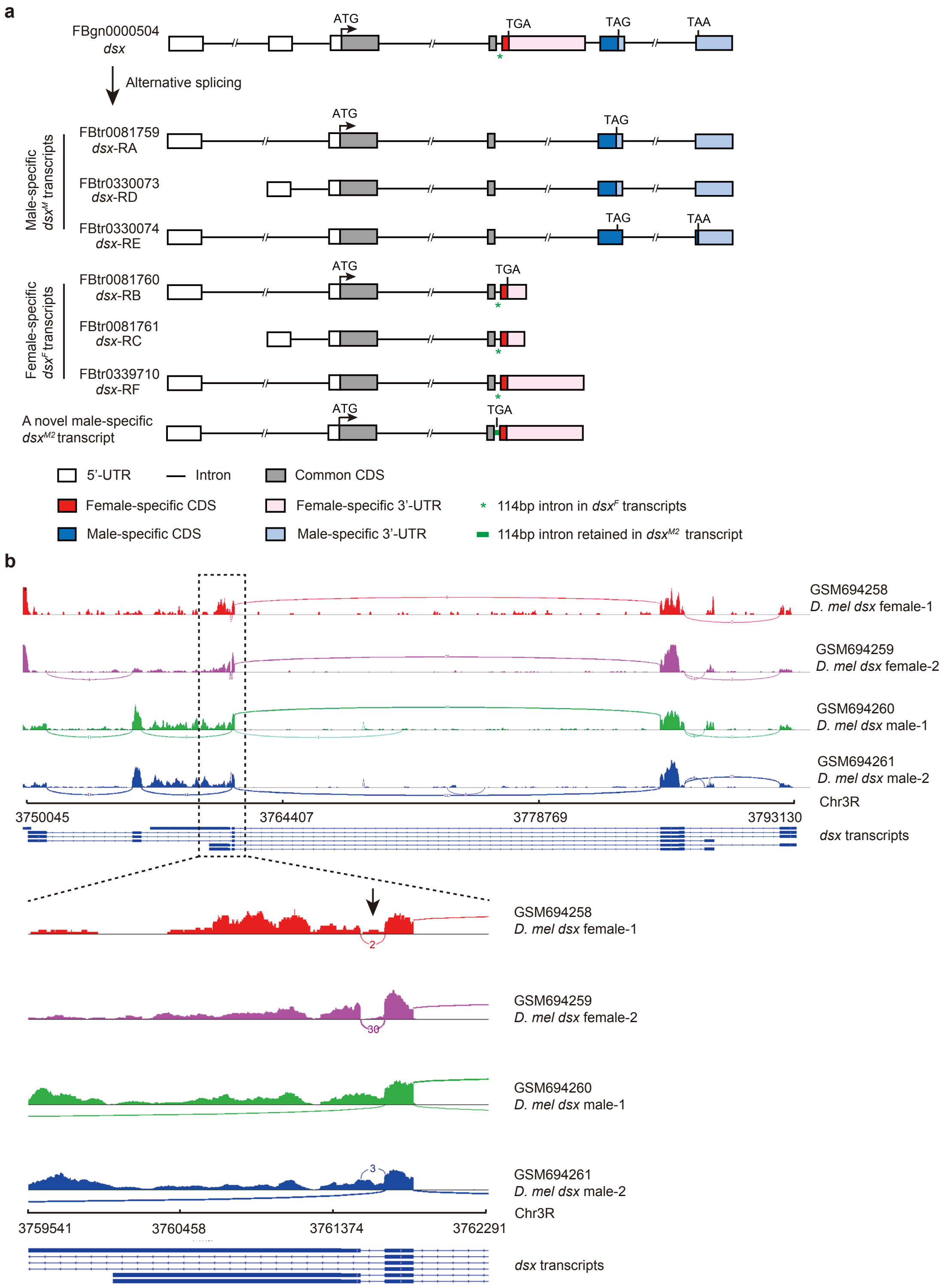

Supplementary Fig. 1: **Overview of *dsx* transcripts in *D. melanogaster* from public datasets. a** Illustration of the six *dsx* transcripts based on the Flybase (http://flybase.org/cgi-bin/gbrowse2/dmel/?Search=1;name=FBgn0000504) and modENCODE databases (http://intermine.modencode.org/release-33/report.do?id=1001006) in *D. melanogaster*. Among the six *dsx* transcripts, *dsx*-RA

and *dsx*-RD encode the known 549 aa Dsx^{M} protein, *dsx*-RE potentially encodes a larger Dsx^{M} protein (572 aa) if reading through the first stop codon in males, while *dsx*-RB, *dsx*-RC and *dsx*-RF encode the known 427 aa Dsx^{F} protein in females. The green asterisk indicates the 114 bp intron in *dsx^{F}* transcripts, and the thick green line indicates the 114 bp intron retention in the male-specific *dsx^{M2}* transcript identified in this work. **b** Sashimi plot visualization of *dsx* splicing events in *D. melanogaster* based on public datasets (https://www.ncbi.nlm.nih.gov/geo/query/acc.cgi?acc=GSE28078). Different colors represent distinct RNA-seq datasets (GSM694258 and GSM694259 for *D. melanogaster* females, GSM694260 and GSM694261 for *D. melanogaster* males). The black dotted box indicates a zoomed region that includes the 114 bp intron (arrow), which is spliced out in female samples but likely to be retained in male samples.

a

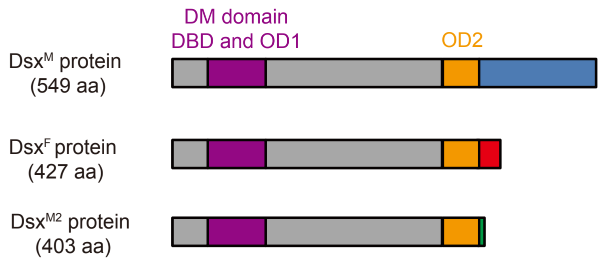

b

Dsx^{M} protein (549 aa)
MVSEENWNSDTMSDSDMIDSKNDVCGGASSSSGSSISPRTPPNCARCRNHGLKITLKGHKRYCKFRYCTCEKCR
LTADRQRVMALQTALRRAQAQDEQRALHMHEVPPANPAATTLLSHHHHVAAPAHVHAHHVHAHHAHGGHHSHHG
HVLHHQQAAAAAAAAPSAPASHLGGSSTAASSIHGHAHAHHVHMAAAAAASVAQHQHQSHPHSHHHHHQNHHQ
HPHQQPATQTALRSPPHSDHGGSVGPATSSSGGGAPSSSNAAAATSSNGSSGGGGGGGGGSSGGGAGGGRSS
GTSVITSADHHMTTVPTPAQSLEGSCDSSSPSPSSTSGAAILPISVSVNRKNGANVPLGQDVFLDYCQKLLEKFRYP
WELMPLMYVILKDADANIEEASRRIEEARVEINRTVAQIYYNYYTPMALVNGAPMYLTYPSIEQGRYGAHFTHLPLTQI
CPPTPEPLALSRSPSSPSGPSAVHNQKPSRPGSSNGTVHSAASPTMVTTMATTSSTPTLSRRQRSRSATPTTPPPP
PPAHSSSNGAYHHGHHLVSSTAAT

Dsx^{F} protein (427 aa)
MVSEENWNSDTMSDSDMIDSKNDVCGGASSSSGSSISPRTPPNCARCRNHGLKITLKGHKRYCKFRYCTCEKCR
LTADRQRVMALQTALRRAQAQDEQRALHMHEVPPANPAATTLLSHHHHVAAPAHVHAHHVHAHHAHGGHHSHHG
HVLHHQQAAAAAAAAPSAPASHLGGSSTAASSIHGHAHAHHVHMAAAAAASVAQHQHQSHPHSHHHHHQNHHQ
HPHQQPATQTALRSPPHSDHGGSVGPATSSSGGGAPSSSNAAAATSSNGSSGGGGGGGGGSSGGGAGGGRSS
GTSVITSADHHMTTVPTPAQSLEGSCDSSSPSPSSTSGAAILPISVSVNRKNGANVPLGQDVFLDYCQKLLEKFRYP
WELMPLMYVILKDADANIEEASRRIEEGQYVVNEYSRQHNLNIYDGGELRNTTRQCG

Dsx^{M2} protein (403 aa)
MVSEENWNSDTMSDSDMIDSKNDVCGGASSSSGSSISPRTPPNCARCRNHGLKITLKGHKRYCKFRYCTCEKCR
LTADRQRVMALQTALRRAQAQDEQRALHMHEVPPANPAATTLLSHHHHVAAPAHVHAHHVHAHHAHGGHHSHHG
HVLHHQQAAAAAAAAPSAPASHLGGSSTAASSIHGHAHAHHVHMAAAAAASVAQHQHQSHPHSHHHHHQNHHQ
HPHQQPATQTALRSPPHSDHGGSVGPATSSSGGGAPSSSNAAAATSSNGSSGGGGGGGGGSSGGGAGGGRSS
GTSVITSADHHMTTVPTPAQSLEGSCDSSSPSPSSTSGAAILPISVSVNRKNGANVPLGQDVFLDYCQKLLEKFRYP
WELMPLMYVILKDADANIEEASRRIEEGKSVDQ

Supplementary Fig. 2 Comparison of Dsx isoforms in *D. melanogaster*. a and **b** The structure (**a**) and amino acid sequences (**b**) of Dsx isoforms. Grey, purple and orange

boxes (**a**) or letters (**b**) indicate common amino acid sequences of all Dsx isoforms, where the purple indicates the DM domain including a zinc-finger DNA binding domain and an oligomerization domain (OD1), and orange indicates another oligomerization domain (OD2). Blue, red, and green boxes/letters indicate Dsx^M, Dsx^F and Dsx^{M2}-specific C-terminus. The *dsx*-RE transcript potentially encodes a larger Dsx^M protein (additional 23 aa: XQYRNVAAAVAAAAAAAVLFVDN) if reading through the first stop codon in males.

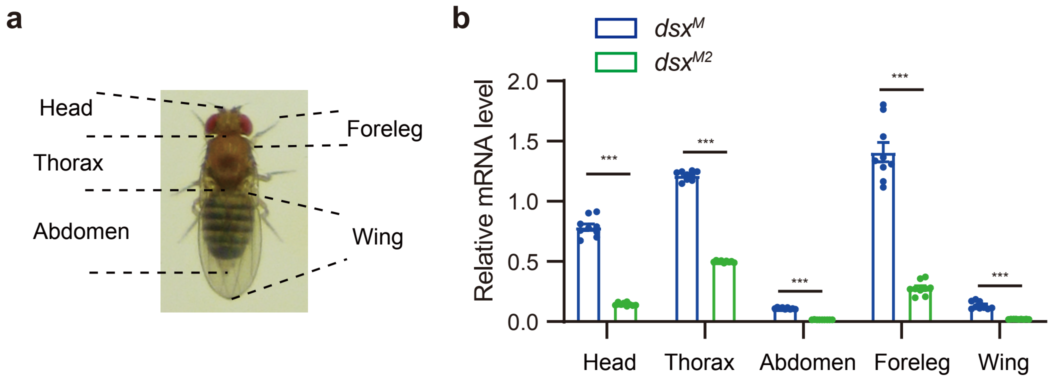

Supplementary Fig. 3 Tissue-specific mRNA expression of *dsx*^{M} and *dsx*^{M2}. a Schematic of tissues used for qPCR. **b** Relative mRNA expression of *dsx*^{M} and *dsx*^{M2} in the head, thorax, abdomen, forelegs, and wings of males. $n = 9$ based on three replicates for each. ***$p < 0.001$, Mann-Whitney U test. Error bars indicate SEM.

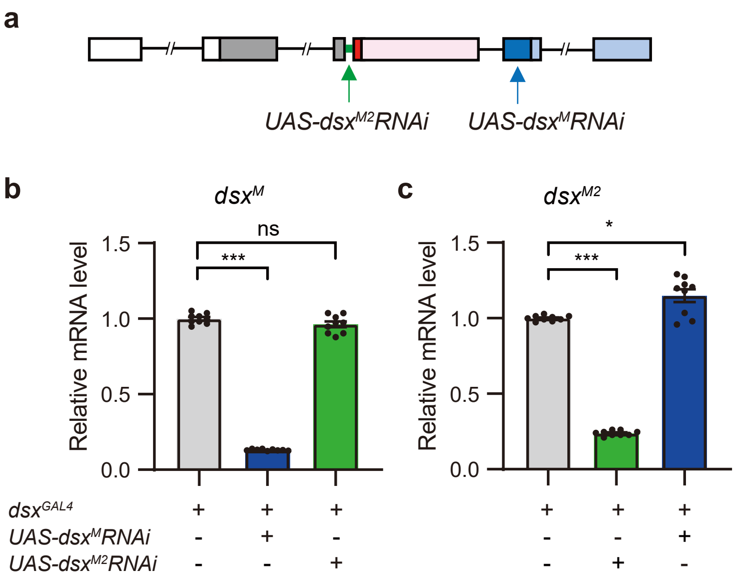

Supplementary Fig. 4 Generation and validation of *UAS-dsx*^{M}*RNAi* and *UAS-*

***$dsx^{M2}RNAi$* transgenic lines.** **a** RNAi targeting *dsx^{M}* and *dsx^{M2}* as indicated by blue and green arrows respectively. **b** and **c** Relative mRNA expression levels of *dsx^{M}* (**b**) and *dsx^{M2}* (**c**) in control and RNAi-mediated males. The *dsx^{M}* or *dsx^{M2}* RNAi knocked down the corresponding *dsx* mRNA efficiently and did not reduce the level of the other transcript. $n = 9$ based on three replicates for each. ns ($p = 0.1648$), not significant, *$p = 0.0333$, ***$p < 0.001$, Mann-Whitney U test. Error bars indicate SEM.

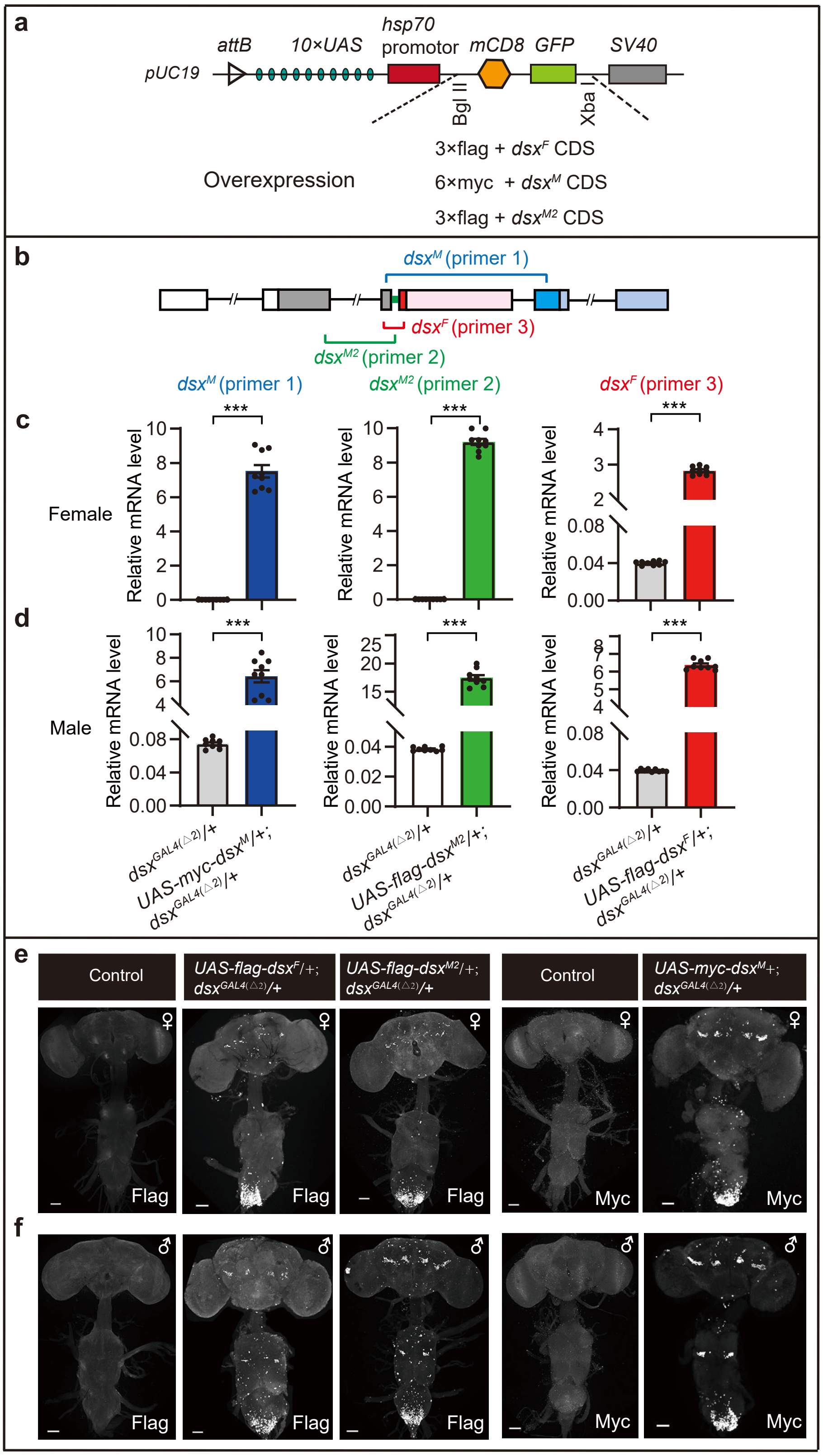

Supplementary Fig. 5 Generation and validation of transgenic flies overexpressing *dsx*^{M}, *dsx*^{M2} or *dsx*^{F}, conjugated with *flag* or *myc* tags. a Diagram of generating transgenic lines overexpressing *dsx*^{M}, *dsx*^{M2} or *dsx*^{F}. **b** Primers against *dsx*^{M}, *dsx*^{M2} or

dsx^F transcripts are indicated. **c** and **d** Relative mRNA expression levels of dsx^M, dsx^{M2} or dsx^F in females (**c**) or males (**d**) with indicated genotypes. n = 9 based on three replicates for each. ***$p < 0.001$, Mann-Whitney U test. Error bars indicate SEM. **e** and **f** Validation of overexpressing dsx^M, dsx^{M2} or dsx^F driven by the $dsx^{GAL4(\Delta 2)}$ through immunostaining with anti-Myc or anti-Flag in the central nervous system of females (**e**) or males (**f**). Genotypes as indicated. Scale bars, 50 μm.

Dsx^{M2} proteins

```
D. mel   MVSEENWNSDTMSDSDMIDSKNDVCGGASSSSGSSISPRTPPNCARCRNHGLKITLKGHK   60
D. sim   MVSEENWNSDTMSDSDMIDSKNDVCGGASSSSGSSISPRTPPNCARCRNHGLKITLKGHK   60
D. moj   MVSEENWNSDTMSDSDMIDSKNDVCGGASSSSGSSISPRTPPNCARCRNHGLKITLKGHK   60
D. vir   MVSEENWNSDTMSDSDMIDSKNDVCGGASSSSGSSISPRTPPNCARCRNHGLKITLKGHK   60
         ************************************************************

D. mel   RYCKFRYCTCEKCRLTADRQRVMALQTALRRAQAQDEQRALHMHEVPPANPAATTLLSHH   120
D. sim   RYCKFRYCTCEKCRLTADRQRVMALQTALRRAQAQDEQRALHMHEVPPANPAATTLLSHH   120
D. moj   RYCKYRYCTCEKCRLTADRQRVMALQTALRRAQAQDEQRSLHMHEVPPPATPAAALLGHH   120
D. vir   RYCKYRYCTCDKCRLTADRQRVMALQTALRRAQAQDEQRSLHMHEVPPPAGATAALLSHH   120
         **** ***** **************************** ********       ** **

D. mel   HHV-------AAPAHVHAHHVHAHHAHGGHHSHHGHVLHHQQAAAAA------------------AAAPSAPA   168
D. sim   HHV-------AAPAHVHAHHVHAHHAHGGHHSHHGHVLHHQQAAAAA------------------AAAPSAPA   168
D. moj   AHHHHVHA--AHSHA------HAH---------HGHAHHGHVLHHQQAAAAAAAAAAAAAGAAPPAQPQ   172
D. vir   GHHHHVHAHAHSHA------HAH---------HGHGHHAHVLHHQQQAAATA-----------VAAAPPPQPP   168
          *      *  **  **     ***         *  **  *******    ***           ** *   *

D. mel   SHLGGSST-----AASSIHGH--------AHAHHVHMAAAAAASVAQHQHQSHPHSHHHHH------Q-----   217
D. sim   SHLGGSST-----AASSIHGH--------AHAHHVHMAAAAAASVAQHQHQSHPHSHHHHH------Q-----   217
D. moj   SHL—NSAHNGTASSLHGHAHAHSHVHHAHMAATSS----VVQQQQQQHHHQQHHQQ------------   223
D. vir   SHLGAAAHNGSAGSLHGHAHAHAHVHHAHMASSAVASVVQQQQQ--HHHQQQQQQHQQHHH   227
         ***          * * ***      * ** ***       * * * * * *

D. mel   NHHQHPHQQPATQTALRSPPHSDHGGSVGPATSSSGGGAPSSSNAAAATSSNGSSGGGGG   277
D. sim   NHHQHPHQHPATQTALRSPPHSDHGGSVGPATSSSGGGAPSSSNATAATSSSGSSGGGGG   277
D. moj   QQQQQQQQQPPHQATLRSPSHSDHGGSVGAATSSSGGGGVGVVGASSSNVAAASSSGSGS   283
D. vir   HSNHQQPQPQPHQASLRSPSHSDHGGSVSAATSSSGGASS---------------SNVATASSSGT----   278
                 *     *   ****  ********   *******                   **  *

D. mel   GGGGSSGGGAGGGRSSGTSVITSADHHMTTVPTPAQSLEGSCDSSSPSPSSTSGAAILPI   337
D. sim   GGGGSSGGGAGGGRSSGTSVITSADHHMTTVPTPAQSLEGSCDSSSPSPSSTSGAAILPI   337
D. moj   ---------AAG-------AGSSGGGISVITSADHHMSTVPTPAQSLEGSCDSSSPSPSSTSGNAVLPI   336
D. vir   ------------AV-------AGAGSGGISVITSADQHMSTVPTPAQSLEGSCDSSSPSPSSTSGNAVLPI   330
                    *      * *******  **  ************************  * ***

D. mel   SVSVNRKNGANVPLGQDVFLDYCQKLLEKFRYPWELMPLMYVILKDADANIEEASRRIEE   397
D. sim   SVSVNRKNGANVPLGQDVFLDYCQKLLEKFRYPWELMPLMYVILKDADANIEEASRRIEE   397
D. moj   SVSSTRK-------NVPLGQDVFLDYCQKLLEKFRYPWELMPLMYVILKDAGADIDEASRRIEE   393
D. vir   SVSSTRK-------NVPLGQDVFLDYCQKLLEKFRYPWELMPLMYVILKDAGADIDEASRRIEE   387
         ***   **      ************************************* * ********

D. mel   GKSVDQ            403
D. sim   GKSLDQ            403
D. moj   GKPKEINHINYFLTN   408
D. vir   GKPT              391
         **
```

Supplementary Fig. 6 Comparison of predicted Dsx^{M2} amino acid sequences in

four *Drosophila* species. Purple sequences indicate the DM domain, orange ones indicate oligomerization domains (OD2), and green ones indicate C-terminus derived from the retained intron sequences. Asterisk indicates identical amino acid sequence in four *Drosophila* species.

a Dsx^{F} specific C-terminal amino acid sequences

```
D. mel  GQYVVNEYSRQHNLNIYDGGELRNTTRQCG
D. sim  GQYVVNEYSRQHNLNIYDGGELRNTTRQCG
D. moj  GQYVVNEYSRQNNLNIYDGGELRNTTRQCG
D. vir  GQYVVNEYSRQNNLNIYDGGELRNTTRQCG
        *********** ******************
```

b Dsx^{M} specific C-terminal amino acid sequences

```
D.mel   ARVEINRTVAQIYYNYYTPMAL------VNGAPMYLTYPSIEQGRYGAHFTHLPLTQICPPTP
D.sim   ARVEINRTVAQIYYNYYTPMAL------VNGAPMYLTYPSIEQGRYGAHFTHLPLTQICPPTP
D.moj   ARVEINRIVAQIYYNYYTPMAIGLHTSAGPMYLTYPSIE-----RYGTHFTHLPLTQIRPPTP
D.vir   ARVEINRIVAQIYYNYYTPMAIGLHTSGAPMYLTYPSIE-----RYGTHFTHLPLTQIRPPTP
         ******* ***************          **********    ***  *********  ****

D.mel   EPLALSRSPSSPSGPS---------AVHNQKPSRPGSSNGTVHSAASPTMVTTMATTSST----PT
D.sim   EPLALSRSPSSPSGPS---------AAHNQKPSRPGSSNGTVHSAASPTMVTTMATTSSTPTPT
D.moj   EPLALSQTPPSPTSAGIPASQHAHQKQSSRPASSNGTPQSAASPAPVTTVTTITAQGH--
D.vir   EPLALSRTPPSPAAESALGVSNPH--QKLSRPASSNGTAHSAASPTLVTTVATITTPGH--
         ******   *  **              *      ***   *****    *****   ***   *

D.mel   LSRRQRSRSATPTTPPPPPPAHSSSNGAYHHGHH----------------------LVSSTAAT
D.sim   LSRRQRSRSATPTTPPPPPPAHSSSNGAYHHGHH----------------------LVSSTAAT
D.moj   HEQ----RSRSG-----TPTTPPPAHSSSNGAYHHQHHHHHHSHHHGQHLVSSTSAAAVAAAAAAT
D.vir   HQQQPRSRSG-----TPNTPPPAHSSSNGAYHH----------HHHHHHGQHLVSSTSAAAVAAAAAAT
               ****     **    ***************                  ***** *
```

c Dsx^{M2} specific C-terminal amino acid sequences

```
D.mel   GKSVDQ
D.sim   GKSLDQ
D.moj   GKPKEINHINYFLTN
D.vir   GKPT
        **
```

Supplementary Fig. 7 Comparison of isoform-specific amino acids of Dsx. a Dsx^{F}-specific C-terminal amino acids are almost identical in the four *Drosophila* species. **b** Dsx^{M}-specific C-terminal amino acids are relatively less conserved in the four *Drosophila* species than those in Dsx^{F}. **c** Dsx^{M2}-specific C-terminal amino acids are relatively short due to the stop codon within the retained intron in the four *Drosophila* species. Asterisk indicates identical amino acid sequence in four *Drosophila* species.

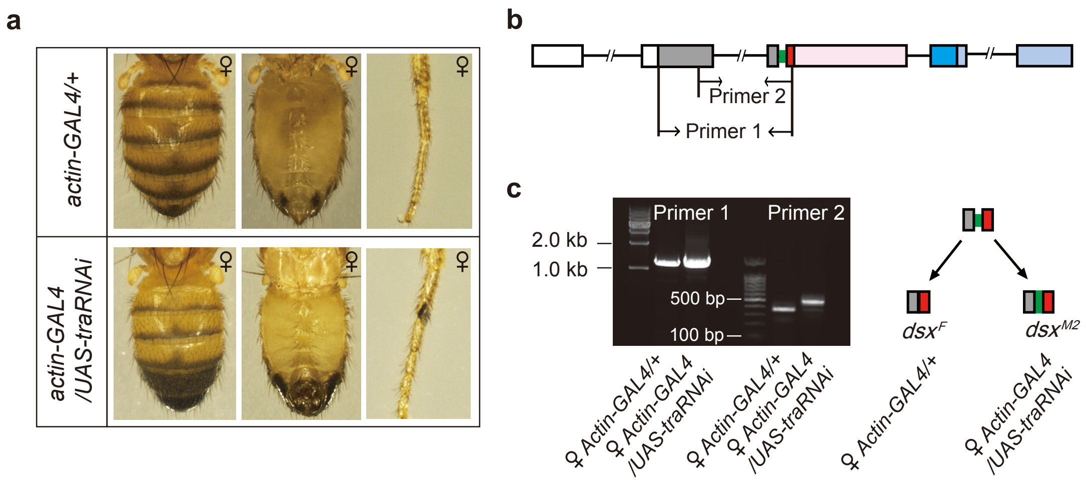

Supplementary Fig. 8 Sex-specific intron retention is mediated by *tra*. a RNAi-mediated knockdown of *tra* driven by the *actin-GAL4* masculinized females. XY and XX flies were discriminated by their eye color (*w-/Y; actin-GAL4/UAS-traRNAi* flies have orange eyes, and *w-/yv*; *actin-GAL4/UAS-traRNAi* flies have wild-type eye color). **b** and **c** Intron retention was observed in females with *tra* knocked down through RT-PCR experiments followed by sequencing.

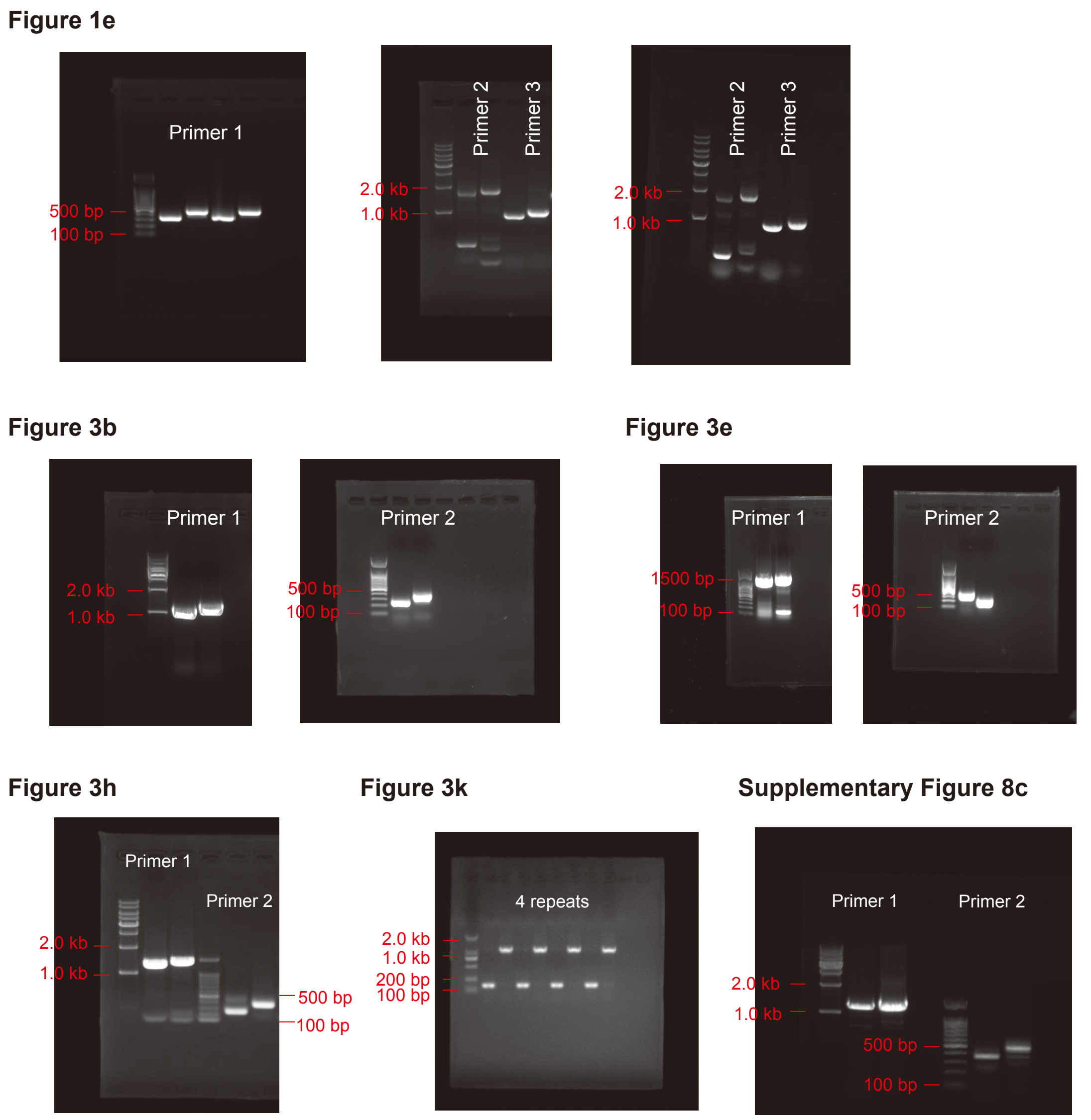

Supplementary Fig. 9 Uncropped gel images corresponding to RT-PCR experiments in this study.

Supplementary Table. 1 Public RNA-seq data shows the 114 bp intron retention in males but not females. These data are a part of supplementary data From Graveley *et al.*[1] (line 25068, Table S27).

as_event_type	intron_retention
gene_name	*dsx*
chr	chr3R

intron-exon_junctions	chr3R_3761375_3761376; chr3R_3761489_3761490
neighboring_constitutive_exons	chr3R_3760200_3761375; chr3R_3761490_3761627
0-24 hr embryos	NA
L1 larvae	NA
L2 larvae	NA
L3 larvae	NA
WPP + 12 hr	12.9
WPP + 24 hr	20
pupae, WPP + 2 days	24.49
pupae, WPP + 3 days	NA
pupae, WPP + 4 days	NA
adult male, eclosion + 1 day	94.74
adult male, eclosion + 5 days	85.94
adult male, eclosion + 30 days	93.94
adult female, eclosion + 1 day	0
adult female, eclosion + 5 days	NA
adult female, eclosion + 30 days	NA

Supplementary references

1. Graveley, B.R. *et al.* The developmental transcriptome of Drosophila melanogaster. *Nature* **471**, 473-9 (2011).
