# Supplementary material for: A male-specific doublesex isoform reveals an evolutionary pathway of sexual development via distinct alternative splicing mechanisms
Source: Commun Biol. 2022 Jul 22;5:728. doi: 10.1038/s42003-022-03664-7 (PMC9307624; doi:10.1038/s42003-022-03664-7)
Supplement: Supplementary file 5 — Reporting Summary [file 42003_2022_3664_MOESM5_ESM.pdf]

nature portfolio

Corresponding author(s): Yufeng Pan

Last updated by author(s): May 25, 2022

Reporting Summary

Nature Portfolio wishes to improve the reproducibility of the work that we publish. This form provides structure for consistency and transparency in reporting. For further information on Nature Portfolio policies, see our Editorial Policies and the Editorial Policy Checklist.

Statistics

For all statistical analyses, confirm that the following items are present in the figure legend, table legend, main text, or Methods section.

n/a	Confirmed	
☐	☒	The exact sample size (*n*) for each experimental group/condition, given as a discrete number and unit of measurement
☐	☒	A statement on whether measurements were taken from distinct samples or whether the same sample was measured repeatedly
☐	☒	The statistical test(s) used AND whether they are one- or two-sided<br>*Only common tests should be described solely by name; describe more complex techniques in the Methods section.*
☒	☐	A description of all covariates tested
☐	☒	A description of any assumptions or corrections, such as tests of normality and adjustment for multiple comparisons
☐	☒	A full description of the statistical parameters including central tendency (e.g. means) or other basic estimates (e.g. regression coefficient) AND variation (e.g. standard deviation) or associated estimates of uncertainty (e.g. confidence intervals)
☐	☒	For null hypothesis testing, the test statistic (e.g. *F*, *t*, *r*) with confidence intervals, effect sizes, degrees of freedom and *P* value noted<br>*Give P values as exact values whenever suitable.*
☒	☐	For Bayesian analysis, information on the choice of priors and Markov chain Monte Carlo settings
☒	☐	For hierarchical and complex designs, identification of the appropriate level for tests and full reporting of outcomes
☒	☐	Estimates of effect sizes (e.g. Cohen's *d*, Pearson's *r*), indicating how they were calculated

Our web collection on statistics for biologists contains articles on many of the points above.

Software and code

Policy information about availability of computer code

Data collection	qPCR: Light Cycler 96; Confocal data: Zeiss 700 or 710; Video recording: Sony FDR-AX40.
Data analysis	Gene or amino acid sequences comparisons: Clustal Omega and LaserGene; confocal images: ImageJ 1.52v; Courtship: LifeSong X; Figure drawing: Prism 8 and Adobe Illustrator CC 2019; Statistics: Prism 8.

For manuscripts utilizing custom algorithms or software that are central to the research but not yet described in published literature, software must be made available to editors and reviewers. We strongly encourage code deposition in a community repository (e.g. GitHub). See the Nature Portfolio guidelines for submitting code & software for further information.

Data

Policy information about availability of data

All manuscripts must include a data availability statement. This statement should provide the following information, where applicable:

- Accession codes, unique identifiers, or web links for publicly available datasets
- A description of any restrictions on data availability
- For clinical datasets or third party data, please ensure that the statement adheres to our policy

All data generated or analyzed during this study are included in the manuscript and its supplementary information files. All other relevant data supporting the findings of this study are available from the corresponding author upon reasonable request. Source data is provided.

Field-specific reporting

Please select the one below that is the best fit for your research. If you are not sure, read the appropriate sections before making your selection.

☒ Life sciences ☐ Behavioural & social sciences ☐ Ecological, evolutionary & environmental sciences

For a reference copy of the document with all sections, see nature.com/documents/nr-reporting-summary-flat.pdf

Life sciences study design

All studies must disclose on these points even when the disclosure is negative.

Sample size	Sample size for tissue staining and imaging ≥5. Sample size for behavioral experiments ≥20. Replicates for PCR ≥3. All samples sizes are indicated in each figure legend. Sample sizes were predetermined based on previous studies in this field (about 20 for behavioral experiments and 5-10 for confocal experiments).
Data exclusions	No data were excluded from the analyses.
Replication	All data presented are representative of at least two independent experiments as indicated in the method part of statistics, and replications were successful.
Randomization	Randomization was irrelevant for the design of this study. All the experimental groups were allocated based on genotypes, and appropriate control groups were assayed in parallel.
Blinding	Investigators were not blinded during data collection and analysis. Experimental manipulation due to implicit bias was not possible without actively tampering with the raw data which are mostly videos, sequencing data and confocal images.

Reporting for specific materials, systems and methods

We require information from authors about some types of materials, experimental systems and methods used in many studies. Here, indicate whether each material, system or method listed is relevant to your study. If you are not sure if a list item applies to your research, read the appropriate section before selecting a response.

Materials & experimental systems

n/a	Involved in the study	
☐	☒	Antibodies
☒	☐	Eukaryotic cell lines
☒	☐	Palaeontology and archaeology
☐	☒	Animals and other organisms
☒	☐	Human research participants
☒	☐	Clinical data
☒	☐	Dual use research of concern

Methods

n/a	Involved in the study	
☒	☐	ChIP-seq
☒	☐	Flow cytometry
☒	☐	MRI-based neuroimaging

Antibodies

Antibodies used	Primary antibodies include Mouse monoclonal anti-Flag (Cat# F1804, 1:500, Sigma–Aldrich), Mouse monoclonal anti-Myc (Cat# M047-3, 1:200, MBL), and secondary Alexa Fluor 488 (Cat# A-21202, 1:500, Thermo Fisher Scientific) and 555 antibodies (Cat# A-31570, 1:500, Thermo Fisher Scientific).
Validation	All the above antibodies (anti-Myc, anti-Flag and secondary antibodies) were commonly and widely used by the field.

Animals and other organisms

Policy information about studies involving animals; ARRIVE guidelines recommended for reporting animal research

Laboratory animals	Drosophila species used: D. melanogaster, D. simulans, D. mojavensis and D. virilis. Genotypes of D. melanogaster were indicated in each figure legends.
Wild animals	The study did not involve wild animals.
Field-collected samples	The study did not involve samples collected from the field.
Ethics oversight	No ethical oversight was required as no vertebrate animals were involved in the research, and no ethical oversight of the experiments was required by our institution.

Note that full information on the approval of the study protocol must also be provided in the manuscript.
